# American Medical Association

**Published:** 1859-07

**Authors:** 


American Medical Association. *Twelfth Annual Meeting.*—Actively instrumental as we were in its foundation, and in giving it form and proportions, when some, whose names are now inscribed on its entablature, held back in doubt bordering on utter disbelief, if not ridicule, of the feasibility of the results hoped for in its inception, we cannot but take a lively interest in all that relates to our great national association of medical men. The recent meeting of this body at Louisville furnishes matter for grave reflection, not so much as relates to what it either has done or failed to do on that occasion, as to its inherent strength and capability to realize the anticipations of its friends. Will it prove itself to be the citadel for the preservation and protection of the great principles of ethics and the fundamental doctrines of medical science, at once a barrier against lawless innovations, and a nursery for the dissemination of new truths? A national medical association is as much a necessity for the body medical of the United States, as a central government is for the body politic of these States, as is, in fact, a sun to be the centre of the planetary and sidereal system. We regard the annual conventions of delegates from the corporate and other medical bodies of the Union as a great step; but it is still only a step, and falls short of meeting the wants and requirements of the profession. These objects can only be fully attained by a permanent body entrusted to carry into effect the decrees of the convention—a body unceasingly vigilant and active in the discharge of the duties devolved in it, and which, even if it should not be endowed with compulsory power, would at least prevent the parties invested with temporary functions, or who have promised consociation, from preferring the plea of forgetfulness or standing upon their dignity, or what they may choose to call reserved rights, but which are only special privileges now obsolete, and no longer compatible with progress and improvement.

A session of three days is quite too short to allow of anything like deliberate discussion of the many important subjects which are brought before the Association. Of these it can do little more than to take the initiative, by receiving propositions in the form of resolutions from its members, and by appointing committees for inquiring into and investigating the matters thus introduced to its notice. Time is not allowed for discussing the hearing and point of any of these matters, so as to ascertain whether a decision could be reached at once, and a vote taken, or a reference be made to a committee. The latter, on its appointment, has no means of learning the sentiments of the Association, or of gleaning useful facts often elicited during a preliminary discussion. In legislative bodies it is customary for the member who either introduces resolutions, or who invokes the appointment of a committee, to place before the house the general merits of the question, and to exhibit the grounds on which he asks for further action, and ultimate decision respecting it. In the Association, a member is afraid to open the subject in this way, knowing that he runs the risk of being prevented from doing it or himself common justice by the ten minutes rule, and probably by a motion of some other impatient member to refer the matter to a committee. Sometimes the reference is proposed in order to stop further discussion, and to procrastinate a decision on a subject which clashes with the prejudices, or the interests of a particular set. In this way a question of the last importance, such, for example, as medical education, may be put off from year to year, with the additional plea of some new light being cast on its merits, or a compromise being effected by the instrumentality of a committee. When, after the expiration of a year, if the committee brings in a report, a not very common thing, by the way, and it is a very long one, the Association has not time to hear it read, and it is referred to the Committee of Publication; so that, in fact, the matter thus passed on, is not a product of the collective wisdom of the great body from which it is supposed to emanate. The paper may be a work of labor and learning, and meet the requirements of the case, or it may be a sophomore essay, which would be reluctantly received in a medical journal the most needy for something to fill its pages. The Association, even if furnished with an abstract of the paper, has not given itself the opportunity of knowing its real character, and the Committee of Publication cannot exercise any critical authority beyond hinting to the writer some gross solecisms in logic or science, which he may or may not receive in a kindly and proper manner.

We are gratified to see that an amendment of this loose working of the Association will be effected for the future, by its having adopted the recommendation of the committee of which Dr. J. B. Lindsly was chairman, viz.: To divide the main body into sections; each section is to take cognizance of a branch of scientific and practical medicine, and after their reading, to discuss the merits of the papers, relating to this branch, which are brought before the Association. The manner in which these sections are to be formed does not appear in the published accounts of the proceedings. If they are large, they will, like all large bodies, be unwieldy and inefficient. In selecting or appointing them, special reference ought to be had to the known capability of the members, and to their being working men.

The American Medical Association met at Mozart Hall, in the City of Louisville, Kentucky, May 3d, 1859. The President, Dr. Harvey Lindsly, called the members to order at eleven o'clock A.M. The Association was welcomed by Dr. R. J. Breckenridge, chairman of the Committee of Arrangements, and by Dr. J. B. Flint, on the part of the Medical Society of Kentucky. The most noticeable resolutions, in the business of the morning, were those directing that every paper, intended for publication in the Transactions, must not only be placed in the hands of the Committee of Publication by the first of June, but must, also, be so prepared as to require no material alteration or addition at the hands of the author. The necessity for a resolution of this kind leads us to infer that great and unwarrantable liberty must have been taken with the Association by the authors of papers, in their presenting them in a crude and unfinished shape, in place of their being carefully prepared and elaborated. It was never intended to make the Association—national as it is in its composition and objects—a school for literary gymnastics, in which untried fledglings should spread their wings for the first time. As, in a measure, a corollary of the first resolution, a second directs that authors of papers shall return their proofs within two weeks after their reception, otherwise they will not be inserted in the volume of Transactions.

The retiring of the President of the former year from the chair was followed by the induction of the President elect, Henry Miller, M.D., of Kentucky. A most discouraging, but at the same time, instructive commentary on the extreme readiness to appoint special committees, without reference to the general attainments and actual readiness of the members who are designated to fill them, was furnished in the fact, that no fewer than twenty-one special committees of last year were unprepared to report at this meeting, and were continued to the next year. Nine of these are announced as being continued or altered—the subjects, as we infer, being the same as before—the composition of some of the committee undergoing a change. Of the standing committees, those on Medical Education and Medical Literature were not prepared to report. In such a state of things, the chairman of the Committee on Nominations, Dr. D. M. Reese, might well call attention to the necessity of some radical change in the mode of appointing committees to prepare treatises on scientific subjects, to be reported at the annual meetings. "It had been seen that on yesterday a large majority of the committees made no reports, and did not even see proper to send in any communication explanatory of delay. The difficulty heretofore has originated in the mode of selection adopted by the Nominating Committee. It has been customary for gentlemen to hand in their names and the proposed subjects on slips of paper, and the committee, without further investigation, have so published in the annual reports. Thus it has happened that appointments have been most injudiciously made, and gentlemen to whom a special duty has been assigned have been found to know less of that than of any other subject. It was therefore hoped that no committee of last year would be reappointed or continued, from which no report had been had and no communication received."

The Association, roused to a sense of an evil which it had always tolerated on previous years, although its patience was never tried to the same extent as now, resolved, unanimously, to instruct the Nominating Committee to act upon the suggestions of the chairman. The latter also stated that there should be some definite expression of disapprobation as to the course of those gentlemen who had volunteered essays, and had their names reported in the newspapers and spread over the land, and then paid no attention to the matter.

After the decided opinion of the chairman of the Nominating Committee, that no committee of last year would be reappointed or continued, from which no report had been had and no communication received, we are surprised to see that, with we believe two or three exceptions, all the delinquent committees have been continued; so much easier is it to discover the mortified part than to apply the cautery. Have greater caution and discrimination been exercised at the late meeting in the appointment of new special committees? No fears can well be entertained of the newly-organized Committee on Medical Education being delinquent next year, as we find that its chairman was at the head of the Nominating Committee, and made the energetic protest against its previous misdoings.

As if the Fates had determined that the volume of Transactions this year should be of the most gracile form, the Committee on Prize Essays could not discover, in any of the four essays presented to it, in time for a careful and thorough examination, the degree and species of merit which should entitle its author to the prize of the Association. Two quite voluminous essays were sent two days only before the meeting, and were very properly excluded from the competition of the present year. The committee asks the Association to determine, in a more precise and formal manner than has yet been done, the terms and conditions of competition and of success in the contest for prizes, for the government alike of contestants and the committee of adjudication.

In order to facilitate, for the future, the dispatch of business, Dr. Harvey Lindsly moved that a select committee of three members be appointed to prepare a system of rules for the government of the Association, the same to be as few in number and as concise and perspicuous as possible, to be reported at the next annual meeting.

Ethical questions of some moment were brought up for consideration and action. Foremost among these was the adoption of an amendment of the constitution, presented last year. It is, that no individual who shall be under sentence of expulsion or suspension from any state or local medical society, of which he may have been a member, shall be received as a delegate to the Association, or be allowed any of the privileges of a member, until he shall have been relieved from the said sentence by such state or local society. Another amendment was also adopted, viz.: No one expelled from this Association shall at any time thereafter be received as a delegate or member except by a three-fourths vote of the members present at the meeting to which he is sent, or at which he is proposed. The Committee on Medical Ethics reported on the complaint made by two medical societies of New Jersey, to the effect that the "New York Medical College" conferred the degree of Doctor of Medicine upon a notorious quack of the name of John F. Dunker, of Newark. The Faculty, through the President of said College, pleaded guilty to the charge, but put in a plea for mitigation of censure, by alleging that they were grossly deceived by false testimonials furnished by said Dunker, and that they have revoked and annulled his diploma, and declared him to be unworthy of patronage or support from authority conferred upon him by this diploma. The committee notices the memorial of the Newark (N. J.) Medical Association as entitled to serious attention, from its showing the negligent manner in which mere verbal and hearsay statements are at times accepted, in place of authentic written testimonials, from individuals presenting themselves as candidates for the honors of the profession at some of the medical colleges of this country. Another matter was referred to the committee, touching a charge preferred by the Dubuque Medical Society of Iowa, against one of its members, which did not call for action, as the difficulty had been adjusted in the State in which the parties reside. In looking at these things, one must feel encouraged at the position occupied by the Association, as a high court of appeal, to which the members of the profession in all parts of this vast country look for the support of their rights and the rebuke of those who infringe the laws of medical ethics.

The following are the committees which made reports on the subjects with which they were charged, viz.:—On Medical Ethics, by John Watson, M.D., of New York; on Governmental Meteorological Reports, by Dr. R. H. Coolidge, U. S. Army; on Criminal Abortion, by Dr. Storer, of Boston; on the propriety of dividing the Association into sections, by Dr. J. B. Lindsly; on Medical Topography and Epidemics, by Dr. Thomas Logan, of California.

The Association, in view of the prevalence and increasing frequency of the crime of procuring abortion, deem it its duty to enter an earnest and solemn protest against such unwarrantable destruction of human life. The subject is presented by the Association to the attention of the several legislative assemblies of the Union, with the prayer that the laws by which the crime of procuring abortion is attempted to be controlled may be revived, and such other measures taken as in their wisdom may seem good. The zealous co-operation of the various State Medical Societies is also invoked, so as to bring the subject before the legislatures of their respective States.

Among the Voluntary Essays was a voluminous one on some of the Changes of the Solids and Fluids in Malarial Fever, by Professor Jones, of Georgia.

Five committees reported, and twenty-one failed to report—a disproportion certainly not complimentary to the Association, nor indicative of any great working ability on the part of its members. We have repeated, after the chairman of the Committee of Nominations, some of the reasons for this lamentable deficiency. Others might be assigned. One which underlies the others has yet to be mentioned; it is the mistaken view of the character of the papers to be laid before the Association. These ought to be original as to the subject, or at least to be fresh contributions on some one or other of the different departments of medical science, illustrated, it may be, by reference to facts and discoveries of a kindred and collateral nature. But long and elaborate treatises on familiar themes, and embracing almost of necessity a good deal of commonplace, are not wanted. The members of the Association turn to volumes on their shelves for information of this kind, and do not feel at all disposed to listen to at the time, or to read afterwards, elementary propositions and descriptions with which it ought to be taken for granted they are familiar. A fanciful notion of the function of an organ is no adequate excuse for introducing a chapter from a received and current work on physiology; and so we would say of pathology and therapeutics. One cannot marvel that the member who is alone on the committee on so vast a subject as the following, should ask for another year to labor at his theme, nor ought we to complain if, with creditable consciousness of the difficulty of his task, he should beg for a still longer period of delay. His subject, as we read it in the published list of the several committees, is—"On the Hygienic Relations of Air, Food, and Water; the Natural and Artificial Causes of their Impurity, and the best methods by which they can be made most effectually to contribute to the Public Health." Here is a full title for a large and comprehensive treatise on hygiene, for the successful execution of which no little preparation, much reading and research, and a fair share of experimental observation are required on the part of the author, who, after he shall have acquitted himself creditably of his task, should offer his work, not to the Association, but to a publisher. Each one of the three great agents of hygiene—air, food, and water—would require the labors of a learned and experienced committee to bring it out in such a shape as should adapt it to the wants and expectations of the Association, for the improvement of the public health. The first part of the subject of the promised report of the committee, or that on the hygienic relations of air, food, and water, has been fully presented in existing works on hygiene. The second, on the natural and artificial causes of the impurity of these three great agents, unless handled with great dexterity, will lead the committee into disquisitions on geology, soil, and meteorology, in relation to the natural causes of the impurity of grains and fruits, and of physiology and rural economy as regards the impurity in animal flesh used for food. The last part of the subject-matter proposed to be examined is alone of sufficient magnitude to task all the knowledge, experience, and perspicuity of the gentleman who, of himself, makes up the committee. It is that also on which the applied science of the day may be brought to bear usefully for the improvement of the public health; and it is the only one that ought to occupy the time and attention of the Association.

We may be rash in our opinion, but it strikes us that if the two gentlemen who constitute the committee on "the Pons Varolii, Medulla Oblongata and Spinal Marrow, their Pathology and Therapeutics," had given the subject even a cursory examination, they would either have shrank from the task devolved on or volunteered by them, we know not which, or have insisted on being associated with the ablest physiologists and pathologists and clinical teachers of the United States; and we do not hesitate to say, that the Association was wanting in its duty in not referring this matter to a committee thus composed. A thorough examination of the subject would task the powers of a Bell, a Rolando, a Lallemand, a Foville, a Flourens, a Hall, a Brown-Séquard, and a Bernard, in its rare and varied anatomico-physiological relations, and of an Abercrombie, an Andral, a Schoenlein, a Romberg, etc. etc. in its pathological and therapeutical bearings. It involves a discussion and solution of some of the most difficult problems in medical science, and it ought not to be approached, for the purposes of a committee report, except by those who have gone fully and faithfully over the whole ground, and who are prepared, while giving us, in brief terms, the conclusions which have been reached by the master-minds who have investigated the subject, to present, at the same time, if not discoveries of their own, at least some pregnant suggestions which may advance us in future inquiries. We write in ignorance of the special qualifications of the two gentlemen who compose the committee on this vast subject. It may be, and we should rejoice to learn, that they have given years to its full and critical study, that they have accumulated from their own observations a large store of pathological changes, and of experiments bearing on the several points included in it, and that they have enjoyed many and rare opportunities for clinical experience by which they have been enabled to note the connection between symptoms and organic lesions and the influence of remedies on both. We might indulge in the same strain of comment on the appointments of some other committees which consist, each, of only one member, and he, we fear, not an "expert," while the subjects to be reported on presuppose prior experience in the mode of investigation and a consequent ability to experiment without being misled by the fallacies which had baffled his predecessors in the same line of inquiry.

A committee, consisting of Dr. J. B. Flint, who had introduced a preamble and resolution on the subjct, and Drs. Bowditch and Shattuck, was appointed to consider in what manner the profession in this country can best participate with their brethren in Great Britain in erecting a monument to the memory of John Hunter. We hope that the projected measure will be as eminently successful in its object as have been the labors of that great and original genius in the advancement of physiology, surgery, comparative anatomy, and simplified pathology, especially in what relates to inflammation and wounds.

As might have been expected, the citizens of Louisville were profuse in their acts of hospitality to the members of the Association, which did, with great sincerity, return its thanks on the occasion. A similar return was made the Committee of Arrangements and the profession of Louisville for their kind and assiduous attention and their hearty welcome.

The Association adjourned to meet at New Haven on the first Tuesday in June, 1860.

We must postpone, for the present, our notice of a meeting recorded under the grandiose heading of "Convention of the Professors of the Medical Colleges of the United States," held at Louisville, on the day preceding that of the Association. Two only of the Eastern schools were represented, viz. Dartmouth College and the Medical Department of Harvard University. Those of New York, Philadelphia, and Baltimore, did not send delegates. The University of Nashville also kept aloof.

The proposals to establish a Board of Censors, in every judicial district of the Supreme Court, who should examine and grant diplomas to all proper members of the Association; also to authorize the Committee on Conference to confer with the State Medical Societies on the subject of preliminary education; and for each State Medical Society to appoint, annually, two delegates to attend the examinations of all candidates for graduation, in participation with and by consent of the colleges, will be noticed more particularly in connection with the Teachers' Convention.

